# Correlation and prognostic accuracy between noninvasive liver fibrosismarkers and portal pressure in cirrhosis: Role of ALBI score

**DOI:** 10.1371/journal.pone.0208903

**Published:** 2018-12-12

**Authors:** Yun-Cheng Hsieh, Kuei-Chuan Lee, Ying-Wen Wang, Ying-Ying Yang, Ming-Chih Hou, Teh-Ia Huo, Han-Chieh Lin

**Affiliations:** 1 Division of Gastroenterology and Hepatology, Department of Medicine, Taipei Veterans General Hospital, Taipei, Taiwan; 2 Faculty of Medicine, National Yang-Ming University School of Medicine, Taipei, Taiwan; 3 Healthcare and Services Center, Taipei Veterans General Hospital, Taipei, Taiwan; 4 Department of Medical Education, Taipei Veterans General Hospital, Taipei, Taiwan; 5 Institute of Pharmacology, National Yang-Ming University School of Medicine, Taipei, Taiwan; Western Sydney University, AUSTRALIA

## Abstract

**Background:**

The role of noninvasive liver fibrosis markers which were developed to evaluate the severity of chronic liver disease remains unclear in cirrhosis.

**Aims:**

To evaluate the correlation between noninvasive markers and hemodynamic parameters and their prognostic performance in cirrhotic patients.

**Methods:**

A total of 242 cirrhotic patients undergoing hemodynamic study were analyzed. The correlations between noninvasive models, including FIB-4, aspartate aminotransferase to platelet ratio index, cirrhosis discriminant score, Lok index, Goteborg University Cirrhosis Index, and albumin-bilirubin (ALBI) score and hemodynamic parameters were investigated, along with their predictive accuracy for short- and long-term survival.

**Results:**

There was a significant correlation between all noninvasive markers and hepatic venous pressure gradient (HVPG), and ALBI score had the best correlation (r = 0.307, *p*<0.001). For the prediction of 3-month and 6-month mortality, serum sodium (sNa) levels had the highest area under curve (AUC; 0.799 and 0.818, respectively) among all parameters, and ALBI score showed the best performance (AUC = 0.691 and 0.740, respectively) compared with other 5 noninvasive models. Of 159 patients with low MELD scores (<14), high ALBI score (>-1.4) and low sNa (<135 mmol/L) predicted early mortality. In the Cox multivariate model, ALBI, MELD, HVPG and sNa were independent predictors of long-term survival.

**Conclusions:**

Among noninvasive markers, ALBI score is best correlated with HVPG and associated with short-term outcome in cirrhotic patients. A high ALBI score and low sNa identify high-risk patients with low MELD scores. High MELD, HVPG, ALBI and low sNa levels are independent predictors of survival. Independent studies are required to confirm our findings.

## Introduction

Portal hypertension (PH) is responsible for complications of liver cirrhosis, such as variceal bleeding, ascites, hepatic encephalopathy and hepatorenal syndrome [1]. Because of these complications, PH represents the main cause of mortality in patients with advanced cirrhosis. Increased intrahepatic vascular resistance and hyperdynamic circulatory alteration both contribute to PH [2].Several studies showed that a reduction in portal pressure determined as the hepatic venous pressure gradient (HVPG) provided effective protection of variceal bleeding [3–5]. Subsequent studies further demonstrated that HVPG predicted the occurrence of decompensation and mortality in cirrhotic patients while a decrease in HVPG improved long-term survival [6,7]. In addition, hemodynamic changes in advanced cirrhosis were found to associate with the development of hepatorenal syndrome [8]. Thus, it is postulated that hemodynamic parameters may provide unique information on the prognosis of cirrhotic patients.

In the past decade, several noninvasive fibrosis markers involving routine laboratory parameters have been developed as an alternative to liver biopsy for evaluating the severity of chronic liver disease, including the FIB-4 index [9], aspartate aminotransferase (AST) to platelet ratio index (APRI) [10], cirrhosis discriminant index (CDS) [11],Lok index [12] and Goteborg University Cirrhosis Index (GUCI) [13].Recently, the albumin-bilirubin (ALBI) score, an alternative measure of liver dysfunction that was initially proposed for use in patients with hepatocellular carcinoma (HCC) [14], has also been found to correlatesignificantly with histological staging in patients with primary biliary cirrhosis [15].As hepatic fibrosis contributes to elevated intrahepatic vascular resistance, various noninvasive markers have been evaluated for their correlation with HVPG in cirrhotic patients. Both the APRI [16] and Lok index [17,18] have shown reliable performance for predicting PH. However, there has been no study evaluating the relationship between portal pressure and ALBI score, and the correlation between noninvasive models and hemodynamic parameters other than HVPG remains unknown. Although noninvasive fibrosis markers have been evaluated for their predictive value with respect to survival in chronic liver disease [17,19,20], the results were inconsistent.The prognostic ability of these models in comparison with the established risk factors such as the model of end stage liver disease (MELD) score, Child-Turcotte-Pugh (CTP) score, serum sodium (sNa) level and HVPG for outcome prediction in cirrhotic patients is still unclear.

This study aimed to investigate the correlation between various noninvasive markers of liver fibrosis and hemodynamic parameters and to assess their prognostic impact on short-term and long-term survival in cirrhotic patients.

## Materials and methods

### Patients

This retrospective cohort study included 242 consecutive adult patients with cirrhosis who had been admitted to Taipei Veterans General Hospital from May 1992 to March 2005for the evaluation of the severity of liver disease and degree of PH. These patients underwent HVPG measurement because it is considered a unique prognostic marker in cirrhosis. The diagnosis of liver cirrhosis was based on characteristic findings including physical stigmata of cirrhosis, biochemical data and image findings [21,22]. Patients with the following conditions were excluded from the study: (1) previous transjugular intra-hepatic porto-systemic shunt placement or portal vein thrombosis; (2) active variceal hemorrhage; (3) hepatic encephalopathy; (4) active infection; (5) HCC; and (6) use of β-blockers or vasoactive drugs upon enrollment. None of the patients with hepatitis B or C had received specific anti-viral treatment (interferon, nucleoside or nucleotide analogues) during the study period.

Medical records were reviewed for data collection including the etiology of liver disease, the presence of esophageal varicesandascites, and transplant-free survival. The severity of ascites was classified into 3 categories: no ascites, response to diuretics, and diuretic-resistant ascites.The presence of ascites was defined by ultrasonography, and the lack of response to diuretics was defined as a mean weight loss less than 0.8 kg over four days under maximal dose of diuretics ora reappearance of grade 2 or 3 ascites within 4 weeks of paracentesis [23].A written informed consent was obtained from each patient before the hemodynamic study was performed.This study complies with current ethical guidelines according to the Declaration of Helsinkiand was approved by theInstitutional Review Board, Taipei Veterans General Hospital, Taipei, Taiwan(No.2017-06-006AC).

### Noninvasive markers of liver fibrosis

Results of laboratory tests performed on the same day before hemodynamic measurement were used to determine of noninvasive markers. These markers, including the FIB-4, APRI, CDS, Lok index, GUCI and ALBI score, were calculated based on the following formulas:

FIB-4 index [9] = age (years) x AST (U/L)/ (platelets (10^9^/L) x alanine aminotransferase (ALT) (U/L)^1/2^)

APRI [10] = AST (/upper limit of normal AST)/platelets (10^9^/L) × 100

CDS is determined as the sum of the following variables and ranges from 0 to 11 [11]: (1) platelet count (10^9^/L): >340 = 0; 280–339 = 1; 220–279 = 2; 160–219 = 3; 100–159 = 4; 40–99 = 5; <40 = 6, (2) the ALT/AST ratio: >1.7 = 0; 1.2–1.7 = 1; 0.6–1.19 = 2; <0.6 = 3, and (3) the international normalized ratio of prothrombin time (INR): <1.1 = 0; 1.1–1.4 = 1; >1.4 = 2. Different points are given and added together according to the values of these parameters.

Lok index [12] = e^(LogOddsLok)^ / (1 + e^(LogOddsLok)^); LogOddsLok = - 5.56 - (0.0089 x platelets(10^9^/L)) + (1.26x AST /ALT ratio) + (5.27 x INR)

GUCI [13] = AST/upper limit of normal AST (U/L) x INR x 100/platelets (10^9^ /L). Upper limit of normal AST equals 45 U/L.

ALBI score [14] = log(bilirubin[mmol/L]) x 0.66)—(albumin[g/L] x 0.085).Patients were stratified into three groups according to previously described cut-offs resulting in three grades: ALBI grade 1 (≤−2.60), grade 2 (>−2.60 to −1.39) and grade 3 (>−1.39).

### Hemodynamic measurement

Patients underwent hemodynamic measurement after an overnight fast. Under local anesthesia, hepatic vein catheterization was performed using a 7F Swan-Ganzthermodilution catheter (Viggo-Spectramed, Oxnard, CA, USA) as previously described [24].Briefly, the catheter was inserted percutaneously using the Seldinger technique into the right internal jugular vein and was then advanced into the right hepatic vein [25], where the free hepatic venous pressure (FHVP) and wedge hepatic venous pressure (WHVP) were recorded with a multi-channel recorder (model 78534C, Hewlett Packard, Palo Alto, CA, USA). The zero reference point was set precisely at 5 cm below the sternum. Under continuous monitoring, confirmation of the wedged position was obtained after injecting a small amount of contrast medium, which demonstrated retention of the contrast medium in the occluded hepatic vein.

The HVPG was obtained by subtracting the FHVP from the WHVP. The catheter was then advanced into the right side of the heart and the pulmonary artery for systemic hemodynamic measurements, which included right atrial pressure (RAP), mean pulmonary arterial pressure, pulmonary capillary wedge pressure, and cardiac output (CO). CO was measured by the thermodilution method [26].The mean arterial pressure (MAP) and heart rate were recorded with an external vital sign monitor. Systemic vascular resistance (SVR) (dyne/s/cm^5^) was calculated as follows: ([MAP–RAP]×80)/CO.An excellent correlation between portal vein pressure and the WHVP has been established in previous studies [27,28].

### Statistical analyses

Chi-squared test or Fisher’s exact test (two-tailed) was used to analyze categorical data, and Mann-Whitney ranked sum test was used for continuous data.Pearson’s correlation analysis was used to estimate the correlation between the MELD, sNa, CTP score, noninvasive markers and hemodynamic parameters. The differences in noninvasive markers with respect to the severity of ascites were assessed using the Kruskal-Wallis test. To assess the abilities of the MELD scores, sNa,HVPG and noninvasive markers to predict the risk of death at 3and 6months, the concordance (c-statistic) equivalent to the area under the receiver operating characteristic curve (AUC) was measured and compared by using the method of Hanley and McNeil [29].

To assess the prognostic predictors of long-term survival, all relevant clinical variables, including age, sex, etiology of cirrhosis, ascites,sNa, HVPG, CTP score, MELD score, and noninvasive markers were entered intounivariate survival analysis and Cox proportional hazards model to determine the adjusted relative risk. Independent prognostic predictors were also evaluated as dichotomous or continuous variables in the Cox model. All statistical analyses were conducted using SPSS for Windows version 12 (SPSS Inc., Chicago, IL) and MedCalc for Windows version 4.2 (MedCalc Software, Mariakerke, Belgium). A *p* value less than 0.05 was considered statistically significant.

## Results

### Patient demographics

Baseline clinicalcharacteristics and laboratory data are shown in Table 1. Patients were predominantly (86%) elderly (mean age: 62±11 years) male. The most common etiology of cirrhosis was viral hepatitis (76%).There were 101 (42%), 86 (36%) and 55(23%) patients belonging to CTP class A, B and C, respectively. Ascites was found in 103 (43%) patients and most patients had esophageal varices (79%). The follow-up period of these patients was up to 13 years (mean: 41 months). During the follow-up period, none underwent liver transplantation due to severe organ shortage in this area.

**Table 1:** Patient demographics.

Demographics	
Age (years)	62 ± 11
Male, n (%)	208 (86)
Etiology of cirrhosis, n (%)	
Hepatitis B	142 (59)
Hepatitis C	41 (17)
Hepatitis B and C	11 (5)
Alcohol	19(8)
NASH and cryptogenic	29 (12)
Ascites, n (%)	
No ascites	139 (57)
Response to diuretics	39 (16)
Diuretic-resistant ascites	64 (27)
CTP score	7±2
Class A/B/C, n (%)	101(42)/86(36)/55(23)
Albumin (g/dl)	3.3±0.6
MELD score	13 ±5
Bilirubin (mg/dL)	2.2±2
INR of prothrombin time	1.4±0.4
Creatinine	1.1±0.6
Serum sodium (mmol/L)	138±4
Platelet (k/cumm)	83±49
ALT (U/L)	50±50
AST (U/L)	67±44
Esophageal varices, n (%)	192 (79)
Hemodynamic parameters	
HVPG (mmHg)	16±5
CO (L/min)	6.70±1.84
SVR (dyne/s/cm^5^)	1142±365
MAP (mmHg)	93±13
Noninvasive markers of liver fibrosis	
FIB-4	9.8±6.9
APRI	2.7±2.2
CDS	8±1
Lok index	0.86±0.17
GUCI	3.8±3.5
ALBI score	-1.8±0.6
Grade 1/2/3, n (%)	34 (14)/132 (55)/76 (31)
Follow-up duration (months)	41±39

Values are presented as mean ± SD or numbers and percentages.

Abbreviations: CTP, Child-Turcotte-Pugh score; MELD, model for end-stage liver disease; INR: international normalized ratio; ALT, alanine aminotransferase; AST, aspartate aminotransferase; HVPG, hepatic venous pressure gradient; CO, cardiac output; SVR, systemic vascular resistance; MAP, mean arterial pressure; APRI, AST to platelet ratio index; CDS, cirrhosis discriminant score; GUCI, Goteborg University Cirrhosis Index; ALBI, albumin-bilirubin.

### Correlation between the MELD score, CTP score, noninvasive fibrosis markers, and hemodynamic parameters

**Fig 1:**
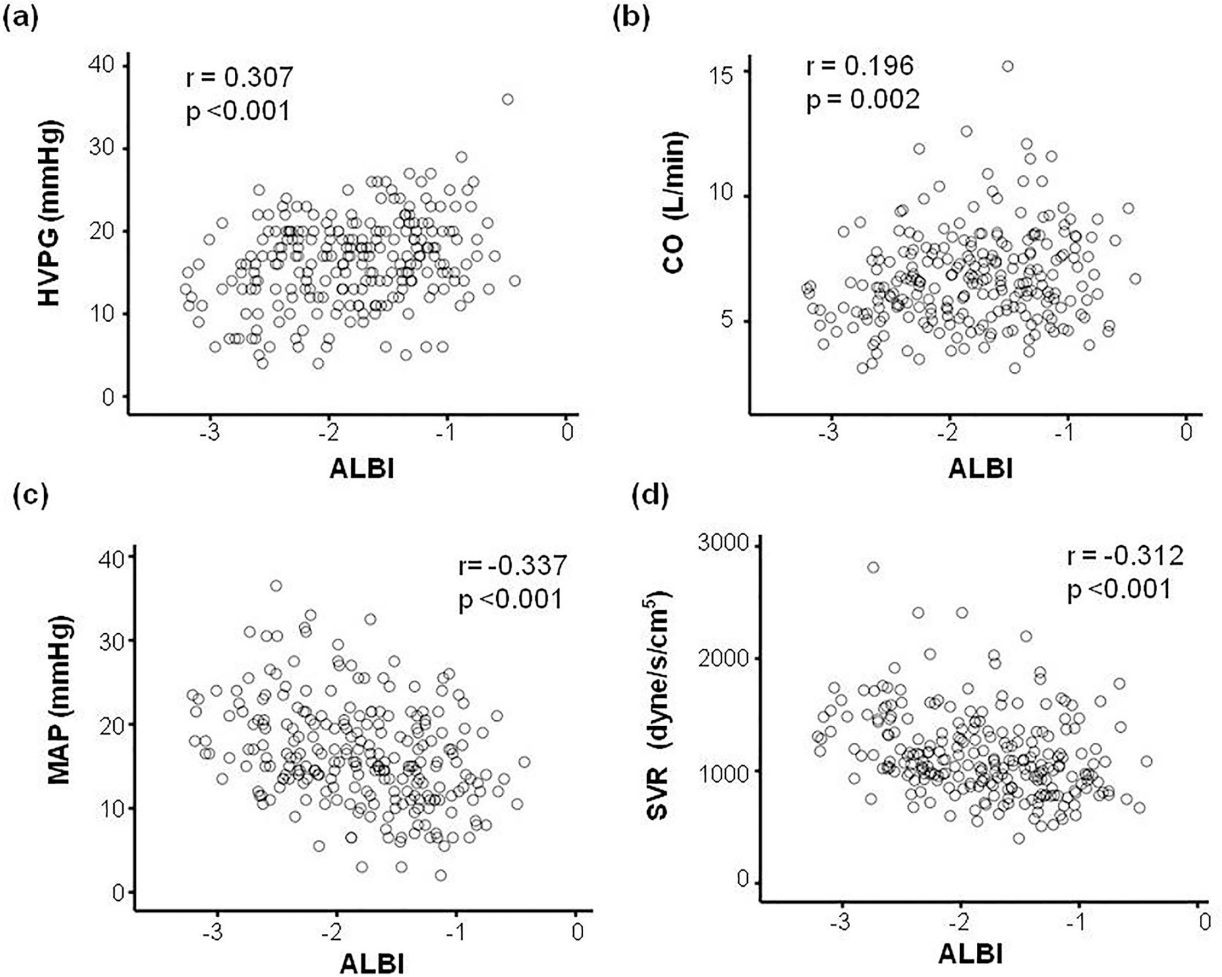
Correlation between albumin-bilirubin (ALBI) scores and hemodynamic parameters in cirrhotic patients. The correlation between ALBI scores and hepatic venous pressure gradient (HVPG, panel A), cardiac output (CO, panel B), mean arterial pressure (MAP, panel C), and systemic vascular resistance (panel D).

There was a positive and significant correlation between the MELD score, CTP score, all noninvasive markers and HVPG (Table 2). sNa had a significant inverse correlation with HVPG.The strongest correlation was observedbetween HVPG and the ALBI score (r = 0.307, *p*<0.001; Fig 1).The AUC for ALBI scores to predict clinically significant PH (HVPG≥10 mmHg) and severe PH (HVPG≥12 mmHg) were 0.721 and 0.671, respectively (*p*<0.001).

**Table 2:** Correlation coefficient (r) amongnoninvasiveliver fibrosismarkers and hemodynamic parameters in cirrhotic patients.

	MELD	CTP score	sNa	ALBI	FIB-4	APRI	Lok index	CDS	GUCI
HVPG (mmHg)	0.205**	0.260**	-0.187*	0.307**	0.270**	0.238**	0.302**	0.261**	0.212**
CO (L/min)	0.140*	0.111	0.002	0.196*	-0.057	0.036	0.143*	0.148*	0.061
SVR (dyne/s/cm)	-0.192*	-0.217**	0.093	-0.312**	-0.066	-0.158*	-0.248**	-0.265**	-0.194*
MAP (mmHg)	-0.196*	-0.279**	0.312**	-0.332**	-0.187*	-0.203*	-0.238**	-0.276**	-0.237**

MELD, model for end-stage liver disease; CTP score, Child-Turcotte-Pugh score; HVPG, hepatic venous pressure gradient; CO, cardiac output; SVR, systemic vascular resistance; MAP, mean arterial pressure; ALBI, albumin-bilirubinscore;FIB-4, fibrosis-4 score;APRI,aspartate transaminase-to-platelet ratio; CDS, cirrhosis discriminant index; GUCI, Göteborg University Cirrhosis Index

*p<0.05

** *p*<0.001

The correlation between the MELD score, CTP score, non-invasive markers and individual hemodynamic parameters were also examined(Table 2). There was a significant and inverse correlation between MAP and the MELD score, CTP score, and noninvasive markers. CO positively correlated with the MELD and ALBI score, whereas SVR was inversely correlated with the MELD score, CTP score, and all noninvasive markers except FIB-4 index. Among these markers, ALBI scores had the best correlation with CO (r = 0.196, *p* = 0.002), SVR (r = -0.312, *p*<0.001) and MAP (r = -0.332, *p*<0.001) (Fig 1).

### Association between noninvasive markers and severity of ascites

The comparison of noninvasive markers of liver fibrosis with respect to the severity of ascites is shown in Table 3. The ALBI score significantly increased with worsening ascites (*p*<0.001). Although the levels of noninvasive markers, except ALBI score, were higher in patients with ascites compared to those in patients without ascites, the difference between patients with ascites that was responsive or resistant to diuretics was not significant.

**Table 3:** Association between the severity of ascites and noninvasive markers of liver fibrosis.

	Severity of ascites			
	No ascites	Respond to diuretics	Diuretics-resistant ascites	*p* value
FIB-4	8.6±5.2	11.5±6.8	11.3±9.3	0.031
APRI	2.4±1.8	3.4±2.4	2.97±2.54	0.057
CDS	7±1	8±1	8±2	0.006
Lok index	0.83±0.17	0.9±0.13	0.9±0.17	<0.001
GUCI	3.2±2.7	4.8±3.9	4.7±4.4	0.010
ALBI	-2±0.6	-1.6±0.5	-1.4±0.5	<0.001

Values are presented as mean ± SD

Abbreviations: APRI, AST to platelet ratio index; CDS, cirrhosis discriminant score; GUCI, Goteborg University Cirrhosis Index; ALBI, albumin-bilirubin.

### Comparison of the predictive accuracy for mortality between the MELD score, HVPG, sNa and noninvasive markers of liver fibrosis at 3 and 6 months

**Fig 2:**
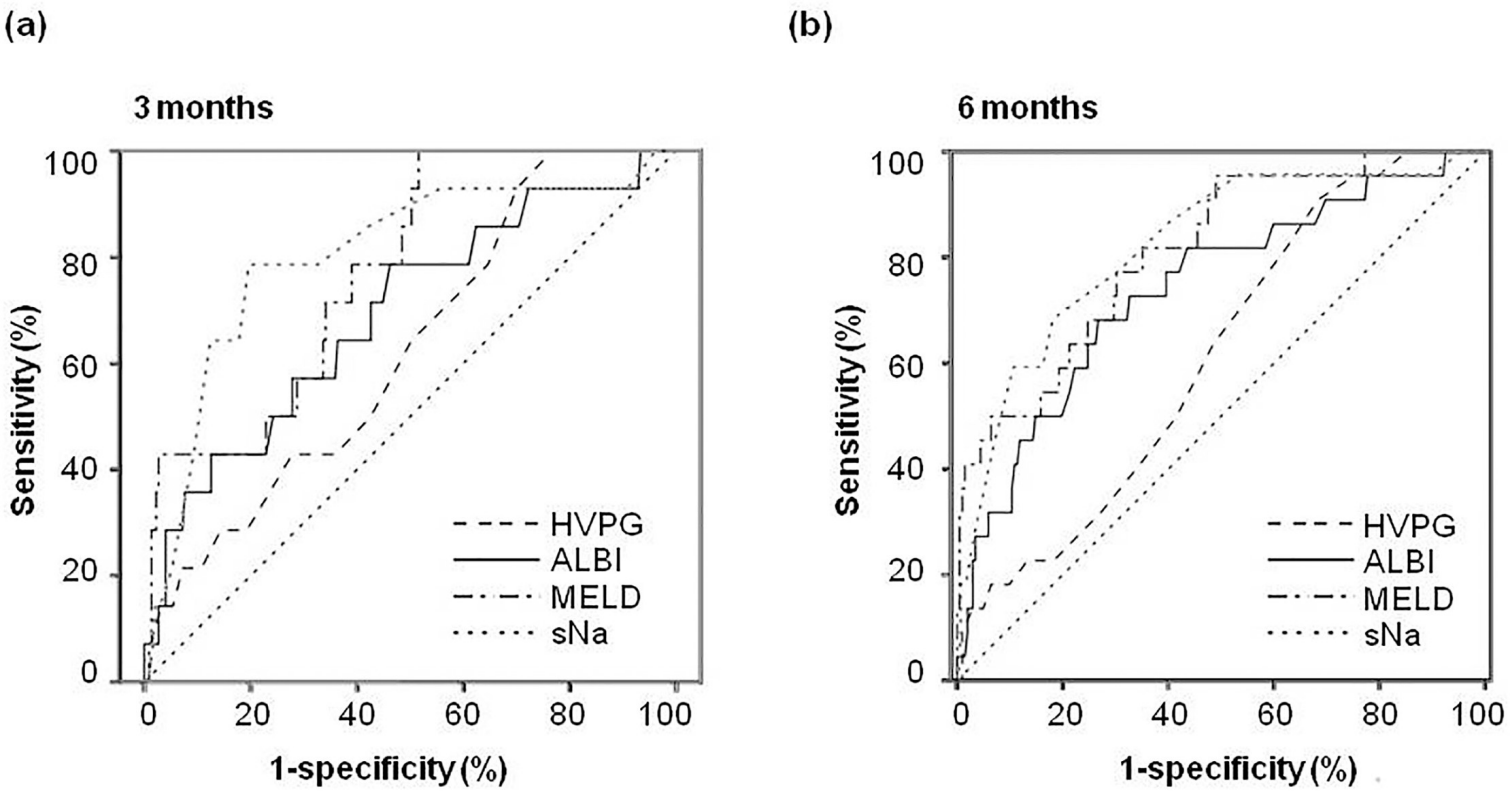
Comparison of the area under curve to predict short-term outcomes. Area under curve of 3-month (panel A) and 6-month (panel B) mortality for model of end-stage liver disease (MELD) score, hepatic venous pressure gradient (HVPG), serum sodium (sNa) and albumin-bilirubin (ALBI) score.

The prognostic performances of noninvasive markers, MELD, HVPG and sNa for predicting 3-month and 6-month mortality are shown in Table 4. Using 3-month mortality as the end point, the AUC was 0.773 for MELD score (*p* = 0.001), 0.626 for HVPG (*p* = 0.115) and 0.799 for sNa (*p*<0.001), respectively. Among the noninvasive markers, only ALBI score significantly predicted 3-month mortality according to the ROC curve analysis (AUC = 0.691, *p* = 0.016).The differences were not statistically significant between the MELD, HVPG, sNa and ALBI score (Table 4 and Fig 2A).At 6 months, the AUC was 0.813 for MELD score (*p*<0.001), 0.615 for HVPG (*p* = 0.081) and 0.818 for sNa (*p*<0.001). The FIB-4 index, CDS, Lok index, GUCI and ALBI score were significantly associated with 6-month mortality by the ROC analysis. Among the noninvasive models, the highest AUC was also observed for ALBI scores (0.740, *p*<0.001).The differences were not statistically significant between the MELD score,sNaand ALBI score.However, both the MELD score and sNa had a significantly higher AUC compared to HVPG (*p* = 0.003 and 0.004, respectively) (Table 4 and Fig 2B). The cut-off values that had the best predictive accuracies for the MELD, sNa and ALBI were determined from the ROC curve. In patients with low (< 14) MELD scores, those with sNa≤135 mmol or ALBI score >-1.4 had significantly higher mortality rates at both 3 and 6 months (Table 5).The AUC for ALBI score in predicting 3-month and 6-month mortality in patients with low MELD scores were 0.738 (p = 0.048) and 0.726 (p = 0.044), respectively. In patients without hyponatremia (sNa>135 mmol/L), those with ALBI scores >-1.4 had higher mortality rate at 3 months (6.8% vs. 1.4%, p = 0.079) and 6 months (12.8% vs 3.6%, p = 0.044).

**Table 4:** Comparison of AUC among MELD, HVPG, sNa and noninvasive fibrosis markers to predict survival at 3 months and 6 months.

	AUC at 3-month	95% CI	*p*	AUC at 6-month	95% CI	*p*
MELD	0.773	0.663–0.882	0.001	0.813	0.720–0.906	<0.001
HVPG (mmHg)	0.626	0.489–0.762	0.115	0.615	0.506–0.724	0.081
sNa (mmol/L)	0.799	0.668–0.930	<0.001	0.818	0.722–0.914	<0.001
FIB-4	0.546	0.370–0.723	0.560	0.643	0.511–0.775	0.028
APRI	0.549	0.370–0.727	0.541	0.593	0.461–0.726	0.151
CDS	0.547	0.396–0.697	0.559	0.695	0.572–0.817	0.003
Lok index	0.625	0.516–0.375	0.116	0.732	0.639–0.825	<0.001
GUCI	0.574	0.397–0.750	0.355	0.640	0.505–0.774	0.032
ALBI	0.691	0.541–0.842	0.016	0.740	0.625–0.854	<0.001

Abbreviations:AUC, area under the curves; CI, confidence interval; MELD, model for end-stage liver disease; HVPG, hepatic venous pressure gradient;sNa, serum sodium levels; APRI, AST to platelet ratio index; CDS, cirrhosis discriminant score; GUCI, Goteborg University Cirrhosis Index; ALBI, albumin-bilirubin

**Table 5:** Prognostic significance of serum sodium and albumin-bilirubin scores for short-term mortality in patients with low (<14) MELD scores.

	3-month, n (%)			6-month, n (%)		
	Death	Alive	*p*	Death	Alive	*p*
sNa≤135 mmol/L	4 (21%)	15 (79%)	0.002	4 (21%)	15 (79%)	0.004
sNa>135 mmol/L	2 (1%)	138 (99%)		3 (2%)	137 (98%)	
ALBI>-1.4	3 (14%)	18 (86%)	0.031	3 (14%)	18 (86%)	0.048
ALBI≤-1.4	3 (2%)	135 (98%)		4 (3%)	134 (97%)	

Abbreviations:MELD, model for end-stage liver disease; sNa, serum sodium levels; ALBI, albumin-bilirubin

### Survival analysis

**Fig 3:**
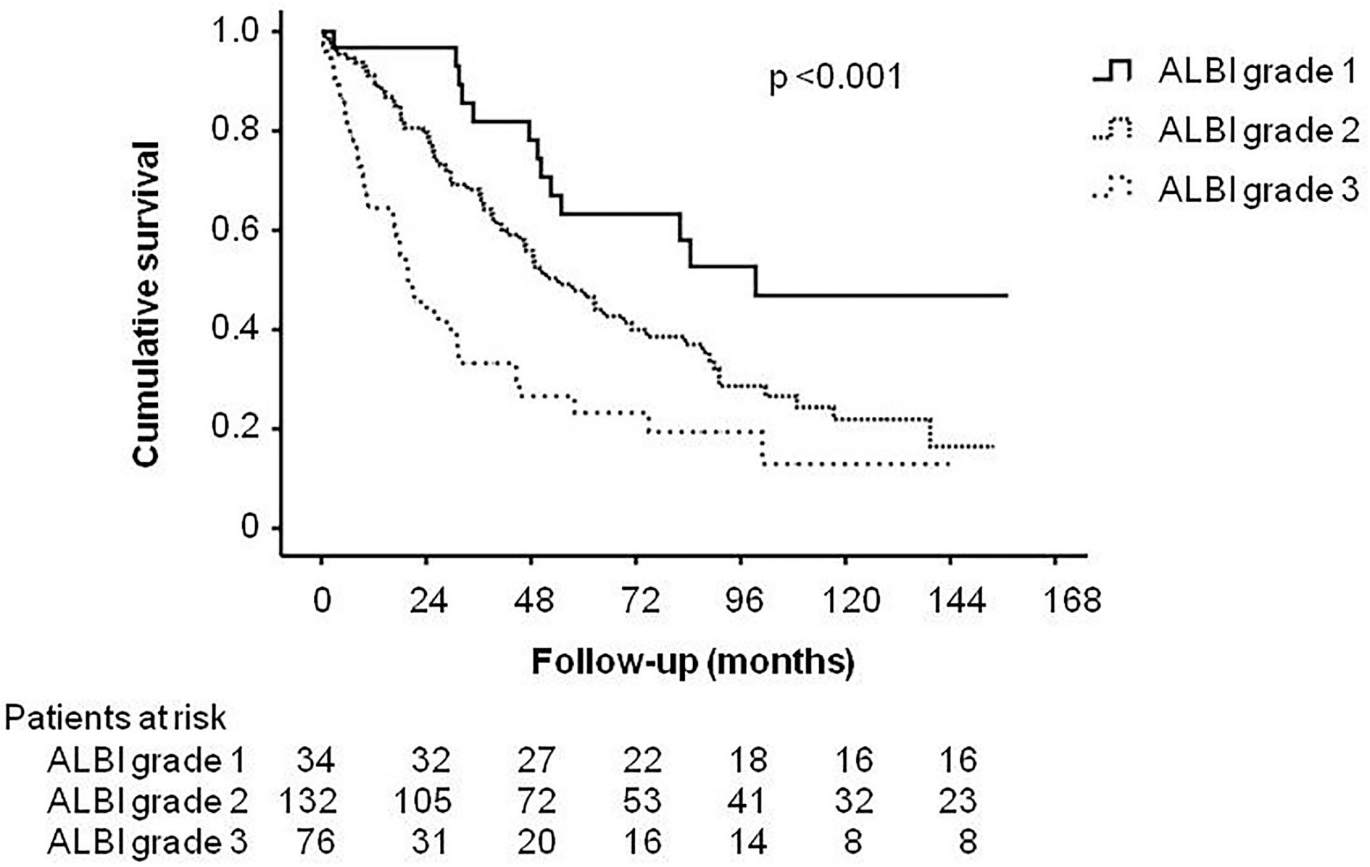
Comparison of long-term survival according to differentALBI grades. ALBI grade 1 patients had a significantly better long-term survival compared to other patient groups.

One hundred and thirty-one (54%) patients died during a mean follow-up period of 41±39 (range: 0.4–157.2) months. The most common causes of mortality were attributable to liver-related diseases(including liver failure, variceal bleeding, spontaneous bacterial peritonitis, hepatic encephalopathy, and hepatorenal syndrome; n = 124, 95%)(Table 6). In univariate survival analysis, male (*p* = 0.007), HVPG >16 mmHg (*p* = 0.001), CTP score >8 (*p*<0.001), MELD score >14 (*p*< 0.001), sNa≤135 mmol/L (*p* = 0.002) and the presence of ascites (*p*<0.001) were factors significantly associated with decreased survival. Among the noninvasive markers, higher ALBI grade (*p*<0.0001), FIB-4 index >8.4 (*p* = 0.005), CDS>7 (*p* = 0.001), Lok index>0.9 (*p*<0.0001), and GUCI>2.7 (*p* = 0.019)were associated with decreased survival. In the Cox multivariate analysis, sNa≤135 mmol/L (hazard ratio [HR]: 1.724, *p* = 0.035), MELD scores >14 (HR:1.829, *p* = 0.005), HVPG >16 mmHg (HR: 1.603, *p* = 0.012) and higher ALBI grade(grade 2 vs grade 1: HR: 1.647, *p* = 0.032; grade 3 vs grade 1: HR: 2.717, *p* = 0.001)wereindependent risk factors predictingpoor long-term survival (Table 7). Patients with ALBI grade 1 had a mean survival of 75.5±46.7 months and patients with ALBI grade 2 and 3 had mean survival of 42.9±37.1 and 22.4±27.5 months, respectively. The survival difference between patients with high and low ALBI grades is shown in Fig 3. When these parameters were treated as continuous variables in the Cox model, there was an additional risk of mortality of 6.9% (*p* = 0.001), 5.6% (*p* = 0.001), and 39.7% (*p* = 0.049) per unit increasein the MELD, HVPG, and ALBI score, respectively. Moreover, there was an additional risk of mortality of 7.5% per unit decrease in sNa levels (*p* = 0.002).

**Table 6:** Causes of death.

Causes	N (%)
Liver related disease	124 (95)
Liver failure	32 (24)
Variceal bleeding	29 (22)
Spontaneous bacterial peritonitis	27 (21)
Hepatorenal syndrome	22 (17)
Hepatic encephalopathy	14 (11)
Pneumonia	3 (2.2)
Soft tissue infection	1 (0.7)
Meningitis	1 (0.7)
Pulmonary hemorrhage	1 (0.7)
Perforated peptic ulcer	1 (0.7)

**Table 7:** Prognostic factors associated with long-term survival in univariate and multivariate analysis.

	Univariate analysis			Multivariate analysis		
	N	Death (%)	p value	HR	95% CI	P value
Age (>65 /≤ 65year-old)	116/126	70/61	0.068			
Gender (male/female)	208/34	121/10	0.007			
sNa (≤135/>135 mmol/L)	51/191	31/99	0.002	1.959	1.090–2.726	0.004
Ascites (yes/no)	103/139	66/65	<0.001			
HVPG (>16/≤16 mmHg)	122/120	76/55	<0.001	1.549	1.072–2.238	0.020
MELD scores(>14/≤14)	83/159	50/81	<0.001	1.844	1.212–2.807	0.004
CTP score (>8/≤ 8)	78/164	49/78	<0.001			
ALBI grade			<0.001			0.008
Grade 2 vs Grade	132/34	72/13		1.647	1.089–3.032	
Grade 3 vs Grade 1	76/34	46/13		2.717	1.728–5.335	
FIB-4 index (>8.4/≤ 8.4)	121/121	72/59	0.005			
APRI (>2/≤ 2)	125/117	72/59	0.053			
CDS (>7/≤ 7)	177/65	105/26	0.001			
Lok index (>0.9/≤ 0.9)	131/111	85/46	<0.001			
GUCI (>2.7/≤2.7)	122/120	73/58	0.019			

Abbreviations: HR, hazard ratio; CI, confidence interval; sNa, serum sodium levels; HVPG, hepatic venous pressure gradient;MELD, model for end-stage liver disease; CTP, Child-Turcotte-Pugh score; ALBI, albumin-bilirubin; APRI, AST to platelet ratio index; CDS, cirrhosis discriminant score; GUCI, Goteborg University Cirrhosis Index.

## Discussion

The present study showed that among the currently used noninvasive liver reserve models, ALBI score had the best correlation with HVPG and other hemodynamic parameters in patients with cirrhosis. Regarding their prognostic value for short-term outcomes, the ALBI score had the highest AUC for predicting 3-month and 6-month mortality compared to those for the other noninvasive markers. In patients with low MELD scores, those with low sNa or high ALBI score had greater short-term mortality. Furthermore, the ALBI as well as MELD score, HVPG and sNa were independently associated with long-term survival in cirrhotic patients.

Noninvasive markers validated in staging liver fibrosis have the advantage of availability and noninvasiveness. Nevertheless, very few of them have been evaluated with respect to their correlation with hemodynamic parameters including HVPG in cirrhotic patients. A previously proposed Fibrotest showed a moderate correlation with HVPG in patients with chronic liver disease with a correlation coefficient of 0.58, but only a weak correlation with HVPG in cirrhotic patients (r = 0.24) [30]. Another study showed that there was a significant correlation between the APRI and HVPG in patients with cirrhosis (r = 0.365) [16]. In our study, we found that all noninvasive markers were significantly correlated with HVPG but the correlation is weak in most models. PH depends not only on the hepatic fibrosis component but also on hemodynamic components which are often associated with splanchnic and portal flow [2]. Although fibrosis markers are related to the increase in intrahepatic vascularresistance consequent to tissue fibrosis, they may not reflect the complex hemodynamic changes characteristicof late PH; thusa fibrosis marker alone may be insufficient to correlate strongly with HVPG.

The ALBI score, a newly prognostic tool involving only two common laboratory parameters of albumin and bilirubin, was initially applied in patients with HCC for assessing the severity of liver dysfunction [14]. The ALBI score was reported to have a positive correlation with HVPG in patients with HCC in a small data set [31]. Our study, which is the first to evaluate the relationship between the ALBI score and hemodynamic parameters in cirrhotic patients, shows that the ALBI score was significantly correlated with HVPG and other hemodynamic parameters, with a higher correlation coefficient compared to that for other fibrosis markers. In addition, the development of ascites is a complication of PH that typically occurs above a HVPG of 12mmHg [32]. The severity of sodium retention increases throughout the natural history of cirrhosis due to the progression of systemic and portal hemodynamic abnormalities and the associated activation of neurohumoral vasoactive systems leading to diuretics-resistant ascites [33].In the present study, although all noninvasive markers were higher in patients with ascites compared to those without ascites, only the ALBI score showed a significant difference between patients with ascites responsive or resistant to diuretics. These findings indicate that the ALBI score is a more unique and specific factor linked to the severity of cirrhosis.

Liver functional reserve is considered as a crucial predictor of mortality in cirrhotic patients. In regard to short-term mortality, the ALBI score was used to predict in-hospital mortality in cirrhotic patients with acute upper gastrointestinal bleeding [34], but its prognostic values compared to the established models is unclear. We investigated the predictive value of the 6 currently used noninvasive models for short-term outcomes, and found that only the ALBI score was significantly associated with 3-month mortality and ALBI score also had the highest AUC for predicting 6-month mortality. There were no significant differences in AUC between the ALBI score, MELD and sNa, suggesting that they have similar predictive accuracies. In addition, the risk of mortality clearly increases with increasing MELD scores, but a substantial portion of patients with initially low MELD scores might have shortened survival [35].Hyponatremia has been proposed as an additional marker to identify patients with a high mortality risk among those with low MELD scores [35]. We demonstrated that a higher ALBI score (>-1.4) was able to predict early mortality in patients with low MELD scores and in patients without hyponatremia. ALBI score was also significantly associated with 3-month and 6-month mortality in patients with low MELD scores. Although the differences were not statistically significant between the MELD score, sNa and ALBI score in predicting short-term mortality, ALBI score can serve as a complimentary prognostic marker. Importantly, ALBI score could help identify high risk subjects in cirrhotic patients with low MELD score or normal serum sodium levels.These results suggest that the ALBI score is not only a prognostic factor of short-term mortality but also a complementary test to predict early mortality among the “low-risk” group.

Regarding the long-term survival, the APRI and FIB-4 have been reported as useful prognostic indicators in patients with chronic hepatitis C [20]. In addition, Lok index was found to predict survival in patients with alcoholic cirrhosis [17].Weevaluated the predictive values of noninvasive markers and the analysis was adjusted by the widely adopted predictorfor prognosis in cirrhosis including MELD, HVPG, sNa and CTP score with a follow-up period up to 13 years. In the univariate analysis, sex, sNa, presence of ascites, HVPG, the MELD score, CTP score, ALBI grade, FIB-4 index, CDS, Lok index and GUCI were significantly associated with survival. In the Cox multivariate model, the MELD, sNa, HVPG and ALBI grade were consistently identified as independent prognostic indicators. When treated as continuous variables, the MELD, sNa, HVPG and ALBI scores were still associated with long-term survival. In a systemic review of prognostic indicators in cirrhosis, serum albumin and bilirubin are the two most prominent individual prognostic variables [36].However, there are limited data on the prognostic information of ALBI score in cirrhotic patients. Chen *et al* reported that ALBI grades were predictors of long-term survival among patients with hepatitis B-related cirrhosis [19]during a 3-year follow-up; however, portal pressure and sNa were not analyzed in the multivariate model. In our study, sNa, MELD score, HVPG and ALBI score are all independent predictors of survival, suggesting that the ALBI score has enhanced prognostic information compared to that for other established indicators.

Our study has some limitations. First, we only included cirrhotic patients who underwent hemodynamic measurement and this may have led to certain selection bias. For example, none of our patients had hepatic encephalopathy at the time of enrollment which has been identified as an important prognostic predictor in cirrhosis.Nearly half (42%) of patients were in CTP class A, suggesting the number of patients with decompensated cirrhosis is relatively low. Thus, our findings may not be readily applicable in the population predominantly with advanced cirrhosis.Second, most patients in the present study had chronic hepatitis B infection as the etiology of cirrhosis and none of them received antiviral treatment at enrollment. The natural course of cirrhosis may vary in patients with different etiologies and specific treatment. Therefore, our results may not be readily applicable in those areas where alcoholism, non-alcoholic fatty liver disease or chronic hepatitis C are major causes of cirrhosis. Third, ALBI scores may be a potential useful predictor of hemodynamic parameters including HVPG in cirrhotic patients, but the correlation between ALBI scores and HVPG in patients without severe portal hypertension needs further studies to evaluate. Last, given the relatively low number of investigated patients, independent cohorts are needed to validate the obtained results.

In conclusion, the ALBI score has the best correlation with hemodynamic parameters and is a feasible marker for short-term outcome prediction in cirrhotic patients. A high ALBI score may identify high-risk patients with low MELD scores and can serve as a complimentary prognostic marker to predict early mortality among this population.Increased ALBI scores, MELD, HVPG, and low sNa are prognostic predictors of decreased long-term survival.Our studydemonstrates the predictive values of ALBI scores in cirrhosis and future prospective cohort with large sample size are needed to validate the findings in the present study and explore the applicability of ALBI score in patients with cirrhosis.

## Supporting information

S1 File The file of the Supporting Information contains de-identified patient research data used in this study. (XLSX)
